# Single Drive Multi-Axis Gyroscope with High Dynamic Range, High Linearity and Wide Bandwidth

**DOI:** 10.3390/mi10060410

**Published:** 2019-06-20

**Authors:** Faisal Iqbal, Hussamud Din, Byeungleul Lee

**Affiliations:** 1 Interdisciplinary Program in Creative Engineering, Korea University of Technology and Education 1600, Chungjeol-ro, Byeongcheon-myeon, Dongnam-gu, Cheonan-si, Chungcheongnam-do 31253, Korea; faisal@koreatech.ac.kr; 2 School of Mechatronics Engineering, Korea University of Technology and Education 1600, Chungjeol-ro, Byeongcheon-myeon, Dongnam-gu, Cheonan-si, Chungcheongnam-do 31253, Korea; hussam@koreatech.ac.kr

**Keywords:** MEMS, inertial sensor, single drive, multi-axis gyroscope, dynamic range, wide bandwidth

## Abstract

This paper presents the design, fabrication, and characterization of a highly sensitive, single drive multi-axis gyroscope. The multi-axis gyroscope allows for a wide bandwidth in all three axes (X, Y, Z) and exhibits high linearity. The fabricated multi-axis gyroscope was fabricated with a structural thickness of 30 µm and packaged at 100 mtorr using wafer level packaging. The fabricated multi-axis gyroscope has a small footprint of 1426 × 1426 µm^2^, making it one of the smallest multi-axis gyroscopes. A custom printed circuit board (PCB) was designed for the evaluation of the multi-axis gyroscope. The experimental results demonstrate that the gyroscope has a high sensitivity of 12.56 μV/dps, 17.13 μV/dps and 25.79 μV/dps in the roll (X-sense), pitch (Y-sense) and yaw (Z-sense) modes respectively. The scale-factor non-linearity of the gyroscope is less than 0.2% for roll and pitch mode and 0.001% for the yaw mode, in the full-scale range of ±1500 deg/s. The multi-axis gyroscope demonstrates an angle random walk of $2.79\;\mathrm{dps}/\sqrt{\mathrm{Hz}}$, $2.14\;\mathrm{dps}/\sqrt{\mathrm{Hz}}$, and $\;1.42\;\mathrm{dps}/\sqrt{\mathrm{Hz}}$, for the roll, pitch and yaw rate with the in-run bias stability 1.62 deg/s, 1.14 deg/s and 0.84 deg/s respectively.

## 1. Introduction

Micro-electro mechanical system (MEMS) vibratory gyroscopes are finding a wide range of applications, including, but not limited to, consumer electronics, inertial navigation systems, image stabilization, and automotive, due to their small size, low cost, light weight, and low power consumption [1,2,3]. 

The principle of the vibratory gyroscope is based on the Coriolis effect, where the drive mode is coupled to the sense mode by the Coriolis force. The two modes, drive and sense mode, can operate either in the mode-match or the mode-split condition. In the mode-match condition, the gyroscope exhibits high sensitivity but limits the bandwidth and dynamic range of the gyroscope. Whereas mode-split condition allows for wide bandwidth and high dynamic range [4,5,6]. 

Until now, high performance single-axis gyroscopes have been developed [7]. To measure the angular rate on all three axes (X, Y, Z), three separate single-axis gyroscopes were used. This kind of configuration results in a large volume and high fabrication costs. Special consideration is required to avoid any misalignment in all three axes. To overcome these issues, discrete, three single-axis gyroscopes fabricated on the same die have been proposed [8], eventually reducing the size and fabrication cost. However, the reduction in size is still limited by the design of individual gyroscopes. Since the power consumption and circuit complexity are high, each gyroscope will operate at different resonant frequencies with its own self-oscillation loop. Recently, single drive multi-axis gyroscopes entered the market [9]. The structures were composed of four masses, providing an in-plane drive motion named “beating heart”. The structures were able to detect angular rate in all three axes simultaneously [10]. 

Compared with single-axis gyroscopes, mechanically designing multi-axis gyroscopes are quite complicated and challenging, as it requires both in-plane and out-of-plane motions. Different design approaches have been used in the literature to improve the performance of multi-axis gyroscopes [11,12,13,14]. An ultra-compact multi-axis gyroscope was reported utilizing the torsional drive mode [11]. The roll (X-sense), pitch (Y-sense) and yaw (Z-sense) modes were decoupled efficiently to reduce cross-axis sensitivity. A mode matched, multi-axis gyroscope, operating at high frequency was reported in [12]. The gyroscope can sustain mechanical shock and vibrations and improve sensitivity. However, the dynamic range and bandwidth of the gyroscope is limited by utilizing the mode match condition. 

Bias errors are also one of the important parameters in the evaluation of vibratory gyroscope performance. These errors arise from the in-phase and quadrature motion of drive mode, coupled with the sense mode [15,16]. Bias errors can be reduced in single-axis gyroscopes, by adopting a symmetrical and decoupled structure [17] and utilizing quadrature cancellation schemes [18,19]. However, in multi-axis gyroscopes, decoupling the drive and sense modes becomes even more complicated. In [20], the drive mode was decoupled from the sense modes by driving the multi-axis gyroscope with secondary auxiliary masses. Driving with the auxiliary masses reduced the in-phase mechanical motion of the drive mode in the sense modes, resulting in high bias stability. 

In this paper, we demonstrate a new single drive multi-axis gyroscope capable of measuring the angular rate in all three axes simultaneously. Four masses are coupled together providing an in-plane drive mode. The multi-axis gyroscope operates in mode-split condition permitting wide bandwidth and high linearity in the full-scale range of ±1500 dps. Following this section, the mechanical design and fabrication process of multi-axis gyroscopes is explained in Section 2. Section 3 presents the evaluation of the multi-axis gyroscope followed by discussion in Section 4. Finally, the paper is concluded in Section 5.

## 2. Multi-Axis Gyroscope Design and Fabrication

### 2.1. Mechanical Design

**Figure 1:**
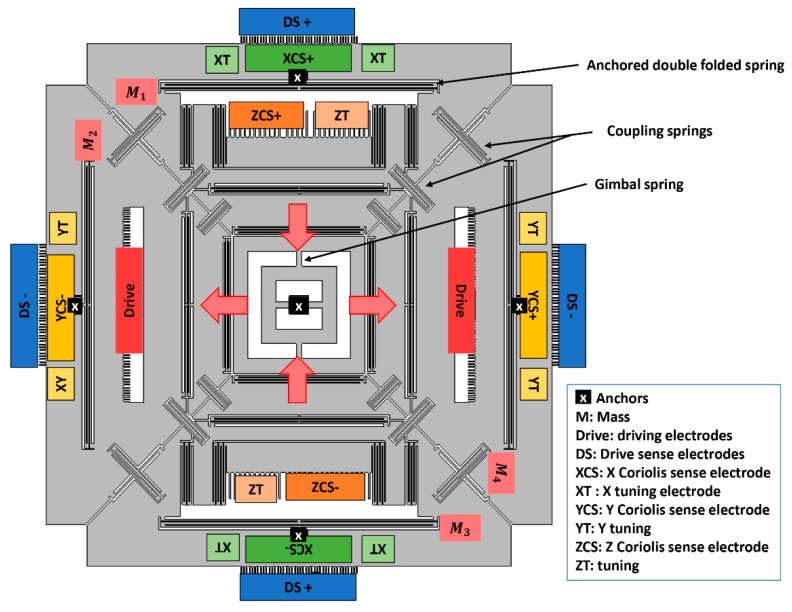
Schematic of the designed single drive multi-axis gyroscope.

**Figure 2:**
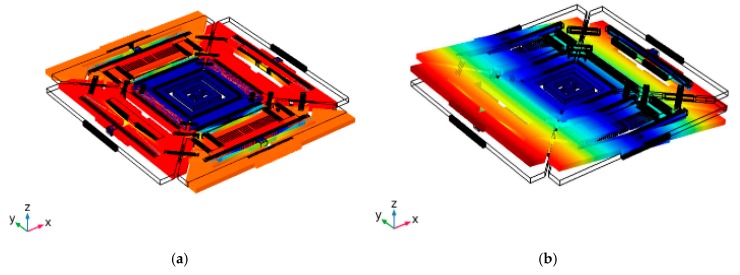

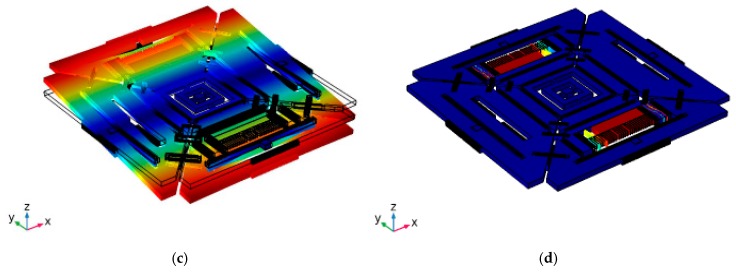
Simulated resonant modes of single drive multi-axis gyroscope (**a**) Lateral drive mode. (**b**) Out-of-plane X sense (Roll mode). (**c**) Out-of-plane Y sense (Pitch mode). (**d**) In-plane Z sense (Yaw mode).

The designed multi-axis gyroscope is shown in Figure 1. The design is based on symmetrically coupled tuning fork architecture. The structure comprises four masses, suspended in the x−y plane relative to the substrate, coupling springs, centrally anchored gimbal spring, and anchored double folded spring. The masses are coupled to each other by coupling springs for the synchronous in-plane drive mode. When two masses, $M_{1}$ and $M_{3}$, move inward, masses $M_{2}$ and $M_{4}$ move outward. This drive scheme was adopted to reduce the slide film damping [21]. The anti-phase drive motion in the x−y plane achieves static and dynamic balancing, resulting in a high quality factor (*Q*) in the drive mode [22]. Each mass is connected to an anchored double folded spring providing an out-of-plane motion. The structure is centrally anchored through a gimbal spring to reduce package stresses. The drive motion, sense motions and coupling mechanism used in the proposed design made it different from the previously reported multi-axis gyroscope presented in the literature [23]. The structure was actuated differentially using comb drive. Differential drive and sensing scheme was adopted to mitigate the effect of linear vibrations and acceleration [3]. The roll and pitch mode can be detected out-of-plane using bottom electrodes differentially, whereas the differential in-plane yaw motion can be detected using parallel plate electrodes. Tuning electrodes are used to tune the sense frequency. The design parameters of single drive multi-axis gyroscope are summarized in Table 1. The modal analysis of the designed structure was carried out using COMSOL Multiphysics. The drive and sense resonant modes are shown in Figure 2.

### 2.2. Fabrication

**Figure 3:**
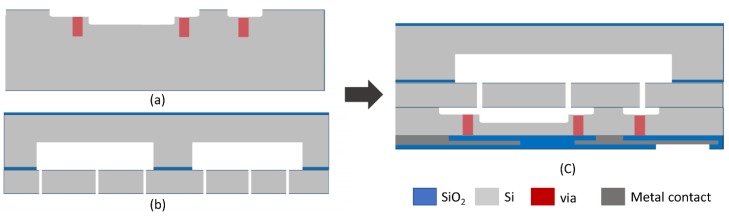
Cross-sectional view of the fabrication process. (**a**) Fabrication of the via wafer. (**b**) Fabrication of cap and device wafer. (**c**) Wafer level bonding and vacuum packaging.

**Figure 4:**
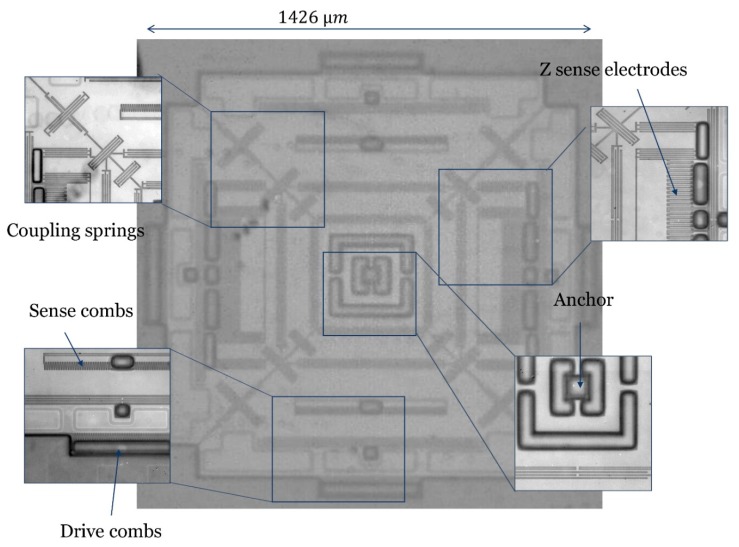
Optical photograph (captured by EMI PHEMOS-1000, Hamamatsu Photonics, Hamamatsu, Japan) of the vacuum packaged single drive multi-axis gyroscope.

The multi-axis gyroscope was fabricated using the three wafer stacked process with a structure layer thickness of 30 μm and packaged at 100 mtorr, exploiting wafer level packaging. The conceptional cross-section is shown in Figure 3. The main fabrication step involves the fabrication of the via wafer and MEMS device wafer. The fabrication starts with the via wafer having 20 μm cavity and 2 μm recess. This is followed by the cap wafer fabrication with a 20 μm cavity. The structure layer is bonded to the cap wafer at 1050 °C (2 stack flow). After polishing and grinding the device wafer to 30 μm, hinges were patterned using lithography and deep reactive ion etching (DRIE) on the device wafer. Finally, the via wafer and device wafer along with the cap were bonded at 100 mtorr vacuum (3 stack process). The optical image of a vacuum packaged multi-axis gyroscope is shown in Figure 4. 

## 3. Characterization 

### 3.1. Resonant Modes

**Figure 5:**
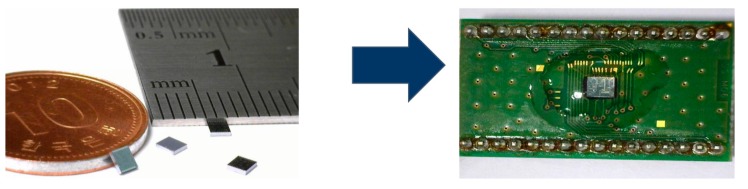
Photograph of diced multi-axis gyroscope and COB.

For a fully functional multi-axis gyroscope, the fabricated multi-axis gyroscope was mounted on a printed circuit board (PCB) as a chip on board (COB) system, as shown in Figure 5. 

The gyroscope was actuated electrostatically by applying a DC voltage of $V_{DC}=5\;V$ with alternating AC voltage of $v_{ac}=100\;\mathrm{mV}$ to the drive electrodes. The sense current was picked up by the charge amplifiers. After the differential amplifier and filter, the frequency responses were measured using the Agilent signal analyzer 35,760 A. The measured resonant frequency of the drive mode was 13,892 Hz, whereas the sense mode’s resonant frequencies were 16,404 Hz (roll mode), 16,214 Hz (pitch mode) and 15,398 Hz (yaw mode). The frequency differences between the drive, roll, pitch, and yaw modes were 2514 Hz, 2322 Hz, and 1508 Hz. 

Theoretically the bandwidth of the gyroscope is approximated as 0.54 times the frequency difference between the drive and sense frequencies [12,24], so the fabricated multi-axis gyroscope allows for the bandwidth of 1200 Hz, 1200 Hz and 800 Hz in the roll, pitch and yaw rate sensing. 

**Figure 6:**
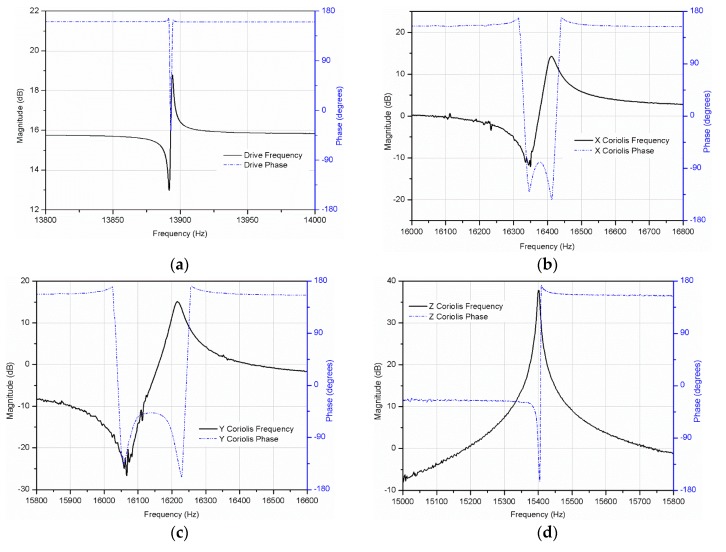
Meaured resonant characteristics of the single drive multi-axis gyroscope (**a**) Drive mode. (**b**) Roll mode. (**c**) Pitch mode. (**d**) Yaw mode.

The quality factor (Q) of the multi-axis gyroscope was measured directly using signal analyzer (Keysight 35670A, Keysight, Santa Rosa, CA, USA) [25]. The Q measured for the drive mode was 12,000, while the roll and pitch mode Q was 500 and the yaw mode Q was 2000. The roll and pitch modes were out-of-plane, which results in low Q compared to the other modes. Figure 6 shows the measured resonant characteristics of the multi-axis gyroscope. 

### 3.2. Angular Rate Response 

**Figure 7:**
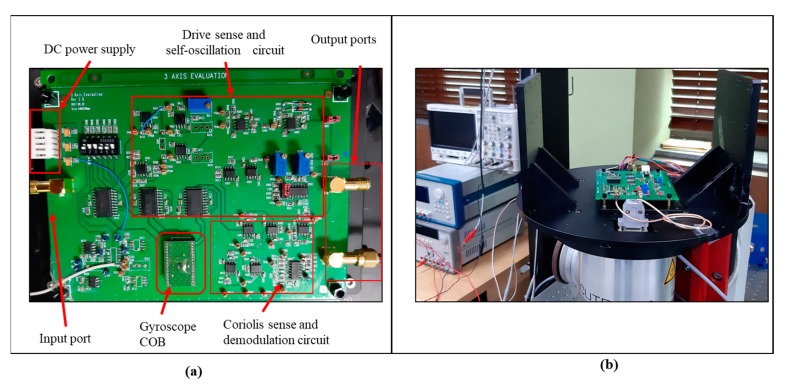
(**a**) Designed PCB for evaluation single drive multi-axis gyroscope. (**b**) Test setup for evaluation of gyroscope.

To measure the angular response of the multi-axis gyroscope, a custom PCB was designed as shown in Figure 7a. The gyroscope was self-oscillated in the drive mode by exploiting the self-oscillation circuit. The amplitude of the drive motion was controlled by utilizing automatic gain control (AGC) in the loop.

**Figure 8:**
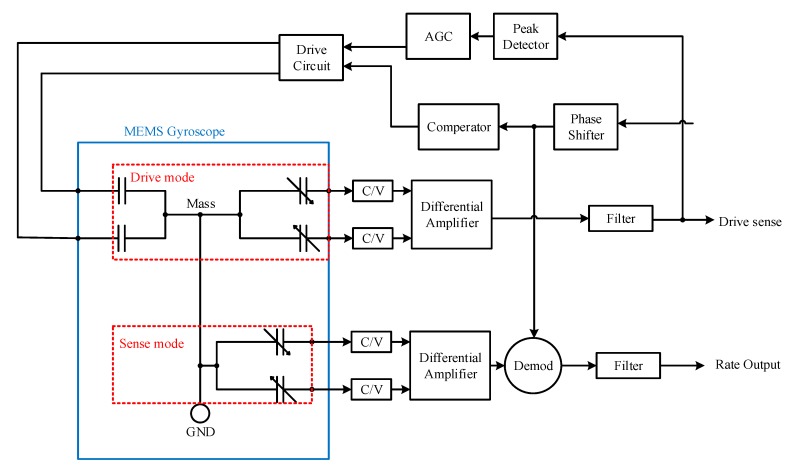
Circuit block diagram.

The open loop Coriolis sense signals were picked by the Coriolis sense electrodes. The sense currents were converted to voltages using charge amplifiers. After the differential amplifier, the sense signal was demodulated using phase sensitive demodulation. A DC rate output was detected after low pass filter. The circuit block diagram is shown in Figure 8. 

**Figure 9:**
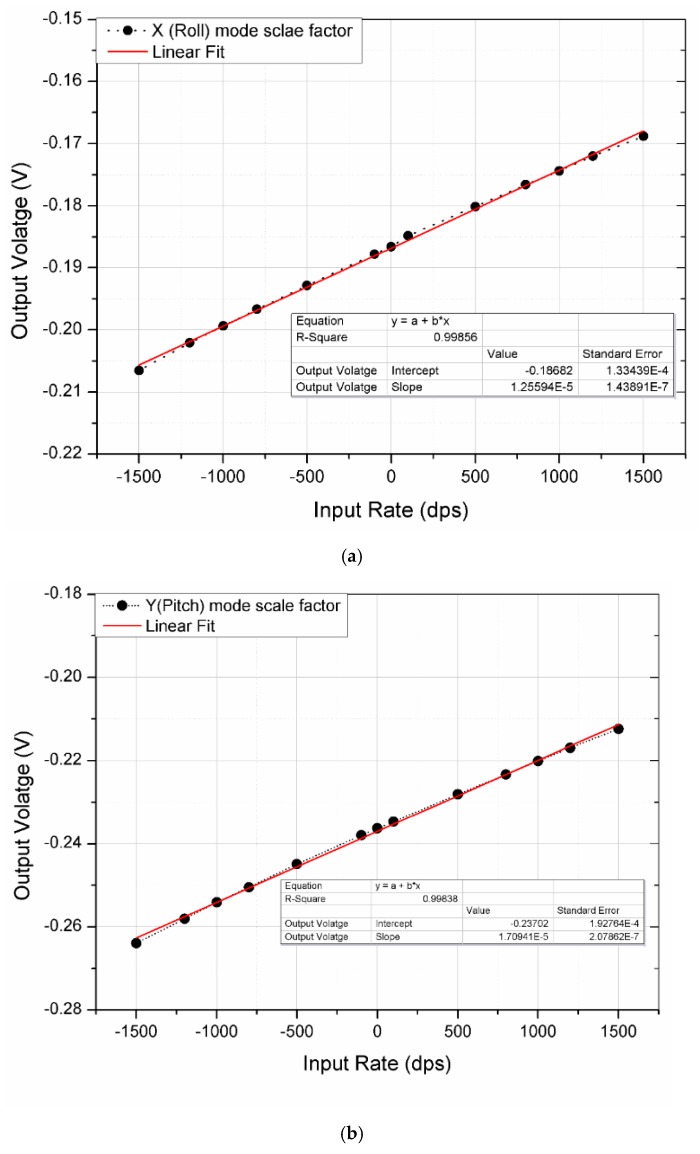

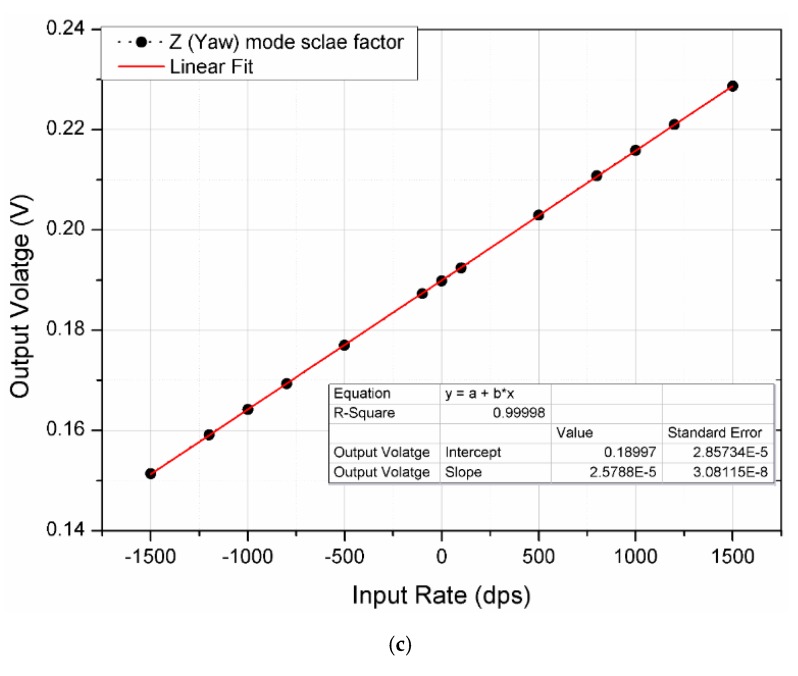
DC response of the multi-axis gyroscope (**a**) Roll. (**b**) Pitch. (**c**) Yaw.

Figure 7b shows the test setup used to measure the angular rate output. The PCB was mounted on a rate table (AC1120S- Acutronic, ACUTRONIC USA Inc., Pittsburgh, PA, USA). The DC output response of the gyroscope to the input rate is shown in Figure 9. The measured sensitivity for the roll, pitch, and yaw rate was 12.56 μV/dps, 17.13 μV/dps and 25.79 μV/dps, for the full-scale range of ±1500 dps. The $R^{2}$-non-linearity for the roll and pitch mode was less then 0.2%, whereas the yaw mode has a non-linearity less than 0.0015%.

### 3.3. Allan Variance 

**Figure 10:**
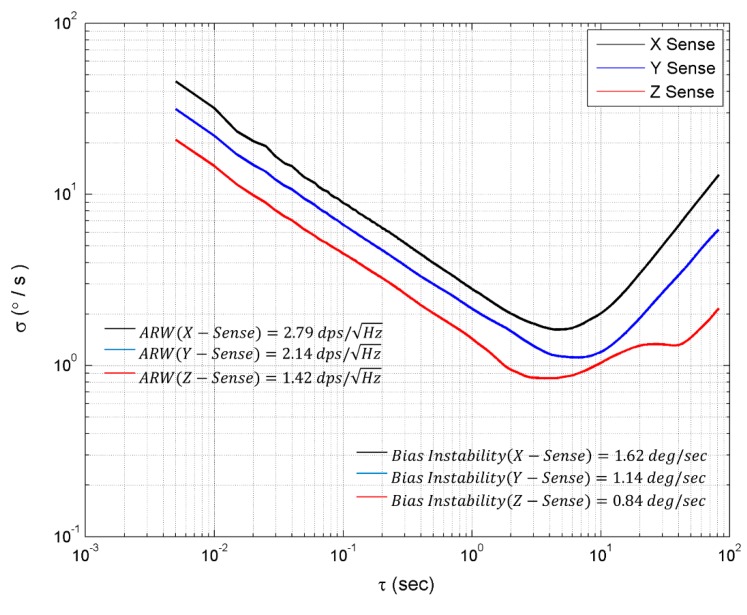
Root Allan variance plot of single drive multi-axis gyroscope.

The noise performance and bias instability of the multi-axis gyroscope was evaluated by the Allan variance plot [26]. The output was collected for 30 min, which was adequate to determine the in-run stability of the multi-axis gyroscope. Figure 10 shows the Allan variance plot for the roll, pitch, and yaw mode. The angle random walk (ARW) determined the noise density, while the bias instability determined the long-term stability of gyroscope. 

The ARW of the designed multi-axis gyroscope was measured by fitting a line at slope τ=1. The measured ARW for roll, pitch, and yaw mode was $2.79\;\mathrm{dps}/\sqrt{\mathrm{Hz}}$,$2.14\;\mathrm{dps}/\sqrt{\mathrm{Hz}}$, and $1.42\;\mathrm{dps}/\sqrt{\mathrm{Hz}}$. The bias instability was measured from the Allan variance plot by fitting a line at slope =0. The multi-axis gyroscope exhibits a bias instability of 1.62 deg/s, 1.14 deg/s and 0.84 deg/s in the roll, pitch, and yaw mode. 

## 4. Discussion 

Table 2 compares the performance of the multi-axis gyroscope with the reported fabricated multi-axis gyroscopes. To avoid external vibrations and acoustic effects, the gyroscope drive mode was deigned above 10 kHz [6,15]. The frequency differences between the drive and sense modes were kept high, about 2 kHz, to allow for wide bandwidth and large dynamic range. The reported gyroscope exhibits high linearity in the full-scale range of ±1500 dps. 

However, the noise performance and long-term bias stability of the proposed multi-axis gyroscope is comparatively high. We believe that this may be due to the following reasons:

(i) The high noise density was due to the custom designed PCB instead of the Readout Integrated Circuit (ROIC). We used a comparator in the self-oscillation loop to maintain the AC signal amplitude, which was the main reason for high noise in the designed PCB [17].

(ii) Drive force coupling and quadrature error: The fabricated multi-axis gyroscope was actuated using a comb-drive to achieve linear motion in the drive mode [27], but they cause an out-of-plane levitation. This out-of-plane levitation couples the drive force to the sense modes resulting in bias errors [28]. The output rate was detected in open loop without any quadrature compensation methods. We believe that the performance can be improved by using a dedicated Application Specific Integrated Circuit (ASIC) along with the quadrature compensation method.

## 5. Conclusions and Future Work

We present the design, fabrication, and characterization of a single drive multi-axis gyroscope. The drive mode resonant frequency of the designed multi-axis gyroscope was designed above 10 kHz to reduce the effect of external mechanical vibrations. The gyroscope was designed by utilizing the mode-split approach with a frequency difference in the drive and sense mode of 2 kHz. This frequency difference allows for wider bandwidth as well as high dynamic range. The fabricated multi-axis gyroscope has a scale factor of 12.56 µV/dps, 17.13 µV/dps, and 25.79µV/dps, with a non-linearity of less than 0.2% in the full-scale range of ±1500.

The angle random walk and bias instability of the gyroscope is relatively high, due to the use of low-cost electronics and evaluation of the design at the PCB level. We are working on the design for a dedicated ASIC and better electronic interface for designed multi-axis gyroscope for better performance. Cross-axis sensitivity was not considered in the current work, which will be evaluated in our future work.

## Figures and Tables

**Table 1:** Design parameters of single drive multi-axis gyroscope.

Parameters	Values			
Mechanical Structure size	1428 × 1428 μm			
Structure thickness	30 μm			
	**Drive**	**Roll**	**Pitch**	**Yaw**
Electrode gap [μm]	8	2	2	1.5
Static capacitance [fF]	748	794	794	582
Measured resonant frequency [Hz]	13,892	16,404	16,214	15,398
Measured Q-factor	12,000	500	500	2000
Bandwidth [Hz]	-	1200	1200	800

**Table 2:** Comparison of the designed multi-axis gyroscope with reported fabricated multi-axis gyroscopes.

Parameters		This Work	Ref [20]	Ref [12]	Ref [11]
Size [mm]		1.4 × 1.4	–	2 × 2	1.2 × 1.2
Resonant Frequency [Hz]	Drive	13,892	27,964	138,058	67,410
	Roll	16,404	25,901	139,140	63,260
	Pitch	16,214	27,115	139,048	63,430
	Yaw	15,398	30,559	138,043	65,000
Scale Factor [µV/dps]	Roll	12.5	28.5	1.40 [pA/dps]	0.12
	Pitch	17.13	57.8	1.2 [pA/dps]	0.09
	Yaw	25.79	19.4	30.5 [pA/dps]	0.3
Measurement Range [dps]		1500	300	150	50
Theoretical Bandwidth (Hz)	Roll	1357.56	1114.02	584.28	2241
	Pitch	1254.96	458.46	534.6	2149.2
	Yaw	814.32	1401.3	8.1	1301.4
Scale factor non-linearity (*R*^2^)	Roll	0.14%	-	-	-
	Pitch	0.15%	-	-	-
	Yaw	0.0015%	-	-	-
Quality factor (*Q*)	drive	12,000	9840	3910	34,000
	Roll	500	927	1181	53,000
	Pitch	500	989	1360	45,000
	Yaw	2000	6744	505	36,000
Angle Random Walk (ARW) $\mathrm{dps}/\sqrt{\mathrm{Hz}}$	Roll	2.79	0.023	0.292	0.06
	Pitch	2.14	0.01	0.357	0.12
	Yaw	1.42	0.036	0.028	0.048
Bias instability deg/s	Roll	1.62	0.043	0.226	0.033
	Pitch	1.14	0.016	0.166	0.039
	Yaw	0.84	0.004	0.041	0.013
